# Comparison of methylation patterns of E6 gene promoter region in the low-risk and high-risk human papillomavirus

**Published:** 2018-12

**Authors:** Ehsan Zafari, Hoorieh Soleimanjahi, Simak Samiee, Hadi Razavinikoo, Zohreh Farahmand

**Affiliations:** 1 Department of Virology, Faculty of Medical Sciences, Tarbiat Modares University, Tehran, Iran; 2 Food and Drug Laboratory Research Center, Ministry of Health and Medical Education, Tehran, Iran; 3 Infectious Diseases Research Center, Golestan University of Medical Sciences, Gorgan, Iran

**Keywords:** Cervical cancer, Methylation, Human papilloma virus, Promoter

## Abstract

**Background and Objectives:**

Cervical cancer is an important cause of death in women worldwide (1, 2). Cancer is a disease that may be caused by many factors that affect gene activity through genetic and epigenetic changes like DNA methylation. DNA promoter methylation contributes to the chromatin conformation that may be repressing transcription of the human papilloma virus type16 (HPV-16), which is prevalent in the etiology of cervical carcinoma. In the present study, we aimed to investigate DNA methylation target sites in promoter region of both high-risk and low risk HPVs.

**Materials and Methods:**

Methylation pattern of E6 promoter in low-risk HPV (type 11) and high-risk HPV (type 16 and 18) was examined by Bisulfite Sequencing PCR (BSP) method.

**Results:**

Based on the results, methylation status of high-risk and low-risk HPV-E6 promoter is different. It was revealed that CpG dinucleotides were unmethylated in type 16 and 18 target sequences, whereas in HPV-E6 type 11 all of CpG dinucleotides were methylated except one of them.

**Conclusion:**

The result suggested that a significant correlation between methylation status and HPV-induced cervical carcinogenesis, and promoter of HPV-16 and 18 E6 has minimal methylation in comparison with low-risk HPV-11.

## INTRODUCTION

Cervical cancer is the third leading cause of cancer deaths among women worldwide (1, 2). The majority of these deaths take place in developing countries (3). This type of cancer is one of the most preventable cancers in the world and screening programs play an important role in reducing the mortality. According to the World Health Organization (WHO) there are no appropriate strategies for prevention including vaccination and screening in many developing nations (4).

High risk human papillomaviruses genome is detectable in more than 97 percent of cases (5). Integration of human papillomavirus (HPV) DNA into the host genome is definitely known as the main cause of cervical cancer (6). High-risk HPV types such as HPV-16 and HPV-18 are involved in cervical cancer and a number of other cancers whereas low-risk types such as HPV 6 and 11 are not generally associated with cancer, but these viruses cause genital warts. High-risk HPV persistent infection in women can induce precancerous lesions in cervix tissue and can progress to cervical cancer after years (7).

Cervical cancer could be avoided by effective screening and management of pre-cancerous lesions, which can be successfully treated (8). The HPV detection in cervical cancer diagnosis can help preventing tumor before it reaches an advanced stage (9). Now in many countries have begun to focus on hrHPV DNA test as primary screening together with cytology (10).

Many studies indicated that E6 and E7 oncogenes of high risk HPV types in addition to other factors are responsible for immortalization of cells and tumor progression (11). Generally, cancer is a disease that may be caused by many factors that affect gene activity through genetic and epigenetic changes which is usually associated with overexpression of oncogenes and the silencing of tumor suppressor genes. DNA methylation is one of the epigenetic mechanisms that cells use to modify function of genes without change in the nucleotide sequence. Except for HPV infection, other factors may increase the risk of cervical cancer that has not been identified well. Cell immortality depend on expression of E6 and E7 oncoproteins that influenced by several agents including transcription factors and epigenetic modifications (12).

HPV genome is a circular, double-stranded DNA which consists of three major regions: early, late, and a long control region (LCR). LCR contain origin of genome replication and several transcription factor binding sites that control viral genome transcription (13). E2 protein is the main regulator of the viral life cycle (14) and modulates host cells especially via its terminal domains interactions. E2 can repress the E6/E7 promoter in a different way. Distinct regulation mechanisms have been described for transcription and translation of E6 and E7 in the high-risk and low-risk HPVs. In low-risk HPVs two distinct promoters drive transcription of E6 and E7 gene while in high-risk HPVs these oncogenes have been transcribed as a “polycistronic mRNA” under the control of a single promoter (14).

Another process that affects viral oncogene expression and tumor progression is methylation of CpG di-nucleotide in viral genome (14). CpG methylation of HPV-16 DNA most distributed in the enhancer and promoter regions that it can be seen at lower levels in advanced stages of cancer. CpG methylation may lead to silencing of tumor suppressor genes and miR-NAs (11). Some studies reported that methylation is a part of HPV life cycle and others suggested that this strategy used in cells to protect itself against virus (13). Actually significantly elevated expression and activity of DNMT1 enzyme can occur as a result of E6 and E7 stimulating activity (15).

Studies show that transient and persistent HPV infections have different methylation pattern. As mentioned, DNA methylation is one of the most important epigenetic mechanisms of oncogene expression. These epigenetic modifications involved in tumor progression and it somehow may be used for diagnostic purposes in the future (16).

Bisulfite treatment of DNA is one of the DNA methylation assays. In this method cytosine residues on single stranded DNA convert to uracils but 5-methyl cytosine retains unaffected. Then methylated and unmethylated cytosines can be distinguished with methods such as methylation-specific PCR.

Regarding the role and importance of papillomavirus E6 oncoprotein in cancer formation and possible correlation between DNA methylation in promoter region and gene expression, we investigated the differences in methylation patterns of high risk and low risk HPV target sequence. In this study methylation pattern of promoter of E6 in low-risk HPV (type 11) and high-risk HPV (type 16 and 18) was examined by Bisulfite Sequencing PCR (BSP) method.

## MATERIALS AND METHODS

### Origin of samples.

HeLa (with 10–50 copies of HPV 18) and CaSki (with 600 copies of HPV 16) cell lines were used as positive control for HPV-16 and HPV-18 respectively. Ca Ski (ATCC^®^ CRL-1550^™^) and HeLa (ATCC^®^ CCL-2) cell lines were obtained from a cell bank (Pasteur Institute of Iran). Confirmed positive clinical samples which included HPV-11, HPV-16, and HPV-18 obtained from Day General Hospital (Tehran, Iran). The DNA of positive clinical samples and control cells were extracted using QIAamp^®^ DNA Mini Kit (QIAGEN, Valencia, CA).

### Bisulfite modifications.

Bisulfite modification of genomic DNA was performed by using the EZ-96 DNA Methylation-Gold Kit (Zymo Research, Orange, CA) according to protocol.

### PCR primers.

**Fig. 1.**
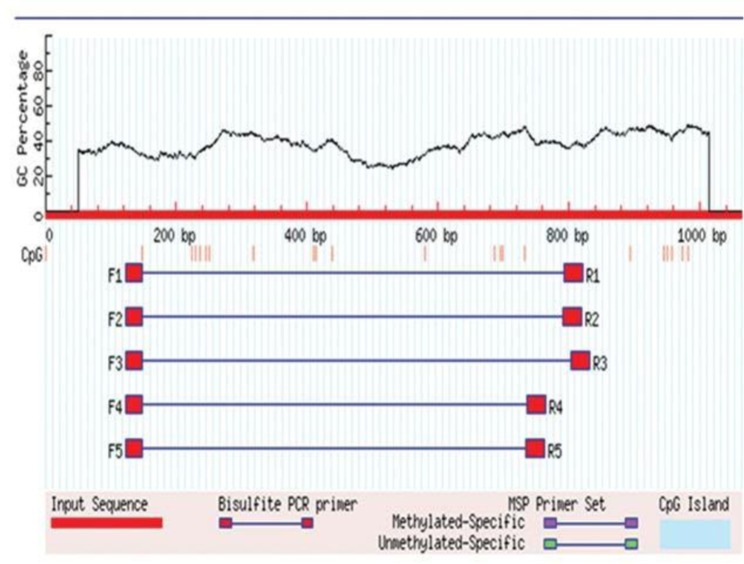
Identification of CpG islands using Met Primer software. Each pink vertical bar represents one CpG site.

Methyl Primer Express v1.0 and Methprimer Softwares were employed for checking the methylation pattern of E6 promoter and designing primer sets (Fig. 1). The characteristics of the primers used for PCR amplifications of E6 promoter, and the PCR conditions are summarized in Table 1.

**Table 1.** List of primers used in PCR amplification of E6 promoter and PCR conditions

**Primer**	**PCR product (bp)**	**PCR conditions**
HPV18_F 5-ATGATTAAGTTGTGTATATATAGTTTATGT-3	309	95°C 15 min, 95°C 30 s, 56°C 30 s, 72°C
HPV18_R 5-TTCAAATACCTCTATAAATTCCAATACTA-3		30 s for 38 cycles and 72°C 15 min.
HPV16_F 5-AAATTGTATATGGGTGTGTGTAAAT_3	248	95°C 15 min, 95°C 30 s, 52°C 30 s, 72°C
HPV16_R 5-TCATATATAATTATTTACAACTCTATACA-3		30 s for 38 cycles and 72°C 15 min.
HPV11_F 5-TGAGTAATTTAAGGTTATATATTTGT-3	330	95°C 15 min, 95°C 30 s, 48°C 30 s, 72°C
HPV11_R 5-ACAACCTTTAAATTCTTATAAACA-3		30 s for 38 cycles and 72°C 15 min.

### PTZR cloning and sequencing.

PCR product of types of 16 and 18 cloned in TA vector. The DH5α strain of *E. coli* competent cells were transformed with confirmed recombinant vectors in Luria-Bertani medium and the plasmids were extracted using intron commercial Kit (DNA_Spin^™^ Plasmid DNA Purification Kit). DNA concentrations were determined by measuring the optical density at 260 nm and the absence of the contaminating *E. coli* DNA or RNA was checked by agarose gel electrophoresis. The presence of the E6 promoter in the constructed vector (PTZ57R/E6 promoter) was determined using PCR (Fig. 6). The DNA sequences of the E6 promoter were fully sequenced.

## RESULTS

**Fig. 2.**
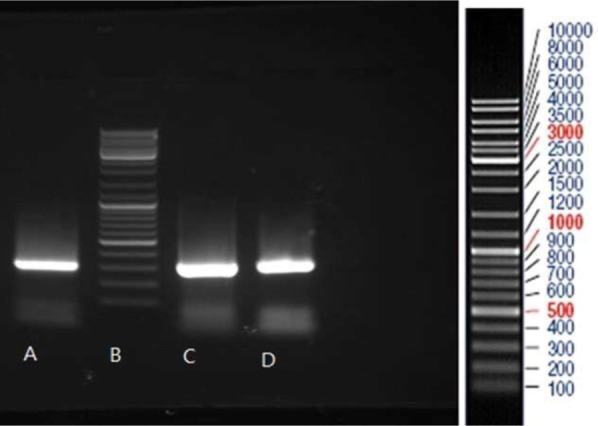
Gel electrophoresis pattern of PCR products of HeLa Cell line (lane A) and two HPV-18 positive clinical samples (lanes C and D) by specific primers amplification. Lane B: DNA marker

**Fig. 3.**
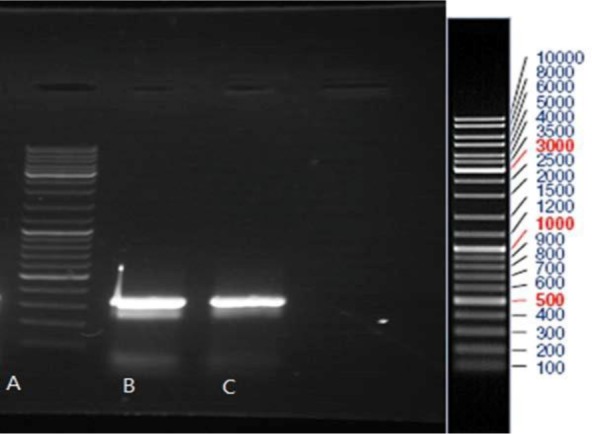
Gel electrophoresis pattern of PCR products of two HPV-11 positive clinical samples (lanes B and C). Lane A: DNA marker

**Fig. 4.**
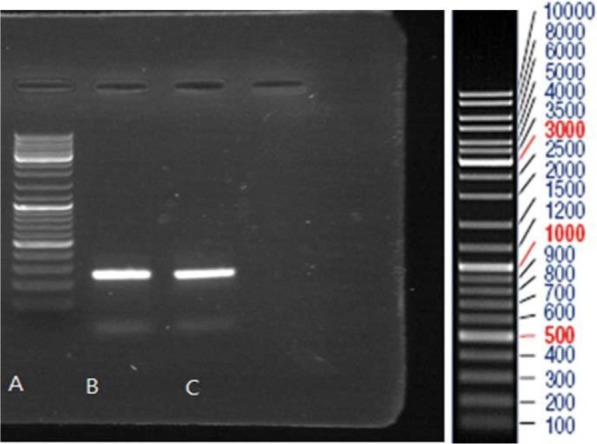
Gel electrophoresis pattern of PCR products of Ca Ski Cell line (lane B) and a HPV-16 positive clinical sample (lane C) by specific primers amplification. Lane A: DNA marker

PCR products were run on agarose gel and the related bands were observed (248 bp for HPV-16, 309 bp for HPV-18 and 330 bp for HPV-11). PCR product of HeLa and Ca Ski cell lines and PCR product of HPV-11, HPV-16, and HPV-18 were run on the gel agarose and observed band was consistent with the expected product length (Figs 2–4).

**Fig. 5.**
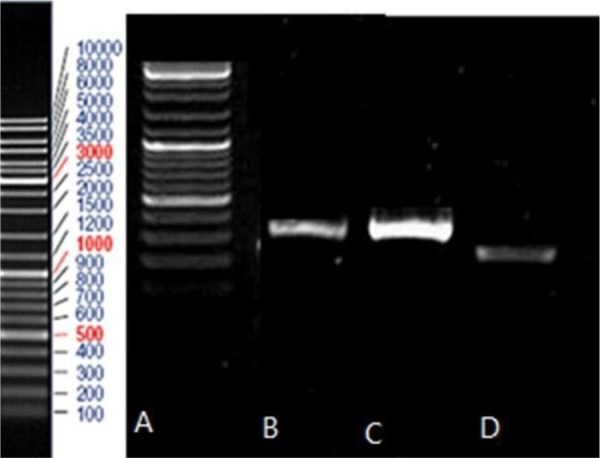
Gel electrophoresis pattern of the colony PCR products. Lane A: DNA marker, Lane B: HPV-18 (309 bp), Lane C: HPV-11(330 bp), Lane D: HPV-16 (248 bp).

**Fig. 6.**
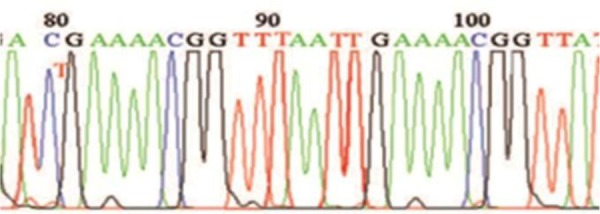
sequencing results of HPV-11 Methylation-specific PCR products. There are three methylated cytosine residues at positions 80, 86 & 101 and only one thymine residue at position 95 in CpG sites after Bisulfite treatment of DNA

**Fig. 7.**
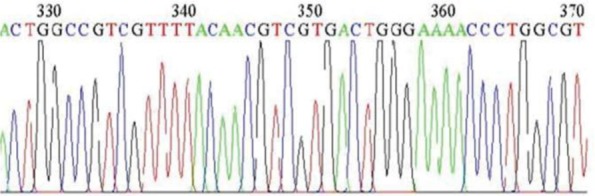
Sequencing results of HPV-16 Methylation-specific PCR products. There are four thymine residues at positions 329, 350, 354 & 365 in CpG sites which were cytosine residues before bisulfite treatment.

After the results of PCR reaction were confirmed by gel electrophoresis, the PCR products of two types of 16 and 18 were cloned in the TA vector, with a positive result of the products of extracted plasmids (Fig. 5). Then products of extracted plasmid and PCR products of HPV-11 and HPV-16 were sequenced. Sequencing results showed significant difference between High-risk and Low-risk HPV pattern. There are 5CpG dinucleotides in promoter region that influenced by methylation. In LR-HPV, 4 CpG dinucleotides from 5CpG dinucleotides were methylated whereas in High-risk HPVs the four of CpG dinucleotides in the promoter region had been were unmetylated (Figs 6, 7).

## DISCUSSION

Although Human papilloma virus (HPV) vaccines played a major role in cervical cancer prevention, but needs to be debated screening of patients (17). Pap smear is used widely for primary screening of cervical cancer but the limitations of cervical cytology and potential side effects from unnecessary treatments are reasons to develop new prevention methods like HPV-DNA based screening especially epigenetic assays (18).

Epigenetic regulation of gene expression including methylation of genome involved in different aspects is important. These epigenetic alterations are involved in main human diseases including cervical cancer progression (19). Although classical CpG islands have not been definitively identified in HPV genome, but there are a significant number of conserved CpG sites which probably play an important role in viral life cycle. The mechanism for these alterations in DNA methylation is not fully understood. It may be a host defense mechanism against viral infection or it probably taken by virus to drive cellular transformation.

Several studies have documented about association between HPV methylome and HPV-related cancers. For example, methylation of HPV DNA especially in L1 gene in CIN2+ cases have different patterns compared to acute or cleared infections (16). It was shown that methylation plays an important role in HPV gene expression and silencing process and different patterns of methylation can be seen in different stages of neoplastic transformation (20).

DNA methylation is a stable characteristic which can be considered as a diagnostic marker and can be performed by using restriction enzyme based differential cleavages pattern of methylated DNA, sodium bisulfite conversion and sequencing and some newer methods, such as methylated DNA immunoprecipitation (Me-DIP) and deep sequencing technologies.

Most of these studies investigated hrHPVs long control region (LCR) methylation pattern which contains E6 and E7 oncogenes promoter. Several studies have reported decreased methylation of LCR is accompanied by cancer progression in HPV16-positive CINs (21). Though, in several other studies increasing methylation within the LCR has been indicated in advanced cervical cancer. These contradictory results may have been caused by the use of different techniques for DNA methylation analysis or different stages of the viral genome in in clinical samples. According to a recent study progressive hypomethylation is accompanied by cervical cancer and promoter hypomethylation is required for activation of important genes in the early stages of carcinogenesis (22).

Since DNA methylation has been identified as a potential biomarker for cervical cancer (23), in this study we examined the promoter region methylation patterns in high-risk and low-risk HPVs. As regards high-risk HPV-16 and HPV-18 have been reported more frequently than other types in cervical cancer, the assumptions were made methylation pattern may have been one of the factors that lead to this types carcinogenicity towards low-risk HPVs. To this end, we compare DNA methylation in promoter region in these two types of HPV. This study showed that promoter CpG islands were hypomethylated in high-risk HPVs (types 16 & 18) compared to low-risk HPV (type 11) and sequencing results showed clear differences in the promoter methylation profiles between high-risk and low-risk HPVs. In type11 only one CpG dinucleotide from 5 CpG dinucleotides was unmethylated and in high-risk HPVs all CpG dinucleotides were unmethylated.

## CONCLUSION

The present study shows differences between two types of low-risk and high-risk HPVs E6 gene promoter methylation patterns and thus between expression levels of E6 oncoprotein and carcinogenic power of these HPV types. HPV promoter methylation profile could be an easy and measurable biomarker to examination of potential carcinogenicity of high-risk HPVs. However, additional clinical sample testing in patients with different grades of CIN are needed to reach a better understanding of DNA methylation pattern as biomarker for different cancer stages.
